# Indecent Publications in Newspapers

**Published:** 1894-03

**Authors:** 


### INDECENT PUBLICATIONS IN NEWSPAPERS.

### MR. RYDER-TAYLOR'S LETTER.

When a man argues from false premises, his conclusions have no value. Mr. Ryder-Taylor, who undertook to defend newspapers against the charge of favoring quacks and fostering abortion, has taken exceptions to certain criticisms of his letter, and has asked for and been given space in this issue for protest.

He assumes that the articles advertised (specifically "Tansy Wafers" and "Cotton Root Pills," universally known as abortifacients, even by the plantation negroes before the war,) have a legitimate use, for which they are advertised,—a false assumption,—and argues that if misused it is not the newspaper's business. He makes it parallel with the misuse of any other drug. Why are they alone of the pharmacopœia selected and offered to women to ' regulate" them? They are the most powerful and most dangerous of the class of emmenagogues, and are never prescribed by physicians. They are intended for abortion, and so it is universally understood.

The complaint against newspapers, and especially the so called religious papers, for advertising objectionable matter, is heard all over the country. It is not alone objected that they advertise abortifacients, but their columns are full of obscene advertisements. Many leading papers contain matter unfit to be seen by any young person, male or female. Witness the "lost manhood" advertisements; and the "book for men only," which offers something to develop and strengthen "certain organs;" what those organs are, not being hard to understand from the picture which accompanies the advertisement, and the announcement that "the triumph of love is fruitful marriage." Witness the 'mother's friend," and "childbirth made easy," advertisements.

These are not only objectionable in themselves, but they are palpable frauds, and any newspaper man who cares to do so can see the fraud on the face of the promises made. The idea of making childbirth easy by rubbing some stuff on the parts concerned in parturition, is too absurd to be a moment entertained by any but those whom it pays to so believe, or pretend to so believe. It is obtaining money under false pretense; and the newspaper, by publishing such promise, is *particeps criminis*. It is not worth while to say that the pains of labor are inseparable from labor, caused by the very nature of the process, the expulsive contractions, and every editor who has a grain of sense or a particle of honesty should know that the promise to annul that pain and make childbirth easy by external application, is a lie—an impossibility; and yet there are thousands of ignorant young women who will catch at it.

The outcry against this feature of newspapers is not confined to any one class or locality; it is not alone the doctors or medical journals who object to and denounce it; but the people are beginning to be aroused. We see that in San Francisco some association of women have begun a crusade against the papers there; and the New York *Medical Record* of February 24th, in commenting on the fact, expresses the hope that women in New York will follow their example. The Texas State Medical Association passed a resolution protesting against it; the pulpit is denouncing it; and we have been assured by an official connected with the postoffice department that while those advertisements are literally a violation of the postal laws, they evade it technically, and that Congress is considering amendments looking to the exclusion of such matter from the mails.

The Amick matter does not pertain to "abortion," the specific matter of our correspondent's letter; but since he has gone out of his way to introduce it, we will say that his statement that Mr. Gurrier has denied it does not disprove the allegation to which Mr. Taylor objects, to-wit: that Amick's advertisements appeared in the Associated Press dispatches. That those advertisements referred to *did appear* in the column devoted to press dispatches, is susceptible of abundant proof; whether Mr. Gurrier sent them as press dispatches or not, or whether he was paid for them or not, or whether the manager of the newspaper placed them in that column, we do not pretend to say; we assert and can show that those advertisements, or similar ones, referring to the Amick matter, did appear in the press dispatch column of at least one paper to our knowledge. It is not to be presumed that it was done for nothing; and the statement of our informant, quoted in our article, and to which Mr. Ryder-Taylor objects, is as much entitled to credence as Mr. Gurrier's denial, and offsets it. It thus becomes a question of veracity between Gurrier and our informant, one of the oldest and best known newspaper men of Texas,—his name will be given if desired,—with the *probabilities* largely in favor of the latter. It is *not probable* that such matter would occupy such space *con amore;* that somebody was paid for it, is certain.

The only way to remedy any evil or institute any reform, is to agitate it. And the medical press should inveigh against the outrage upon decency that newspapers are constantly perpetrating by the publication of indecent and fraudulent advertisements, until relief is obtained in some way.

